# Global phylogeography of a pantropical mangrove genus *Rhizophora*

**DOI:** 10.1038/s41598-021-85844-9

**Published:** 2021-03-30

**Authors:** Koji Takayama, Yoichi Tateishi, Tadashi Kajita

**Affiliations:** 1 Department of Botany, Graduate School of Science, Kyoto University, Kitashirakawa Oiwake-cho, Sakyo-ku, Kyoto, 606-8502 Japan; 2 Faculty of Education, University of the Ryukyus, Senbaru 1, Nakagami-gun, Okinawa, 903-0129 Japan; 3 Iriomote Station, Tropical Biosphere Research Center, University of the Ryukyus, 870 Uehara, Taketomi-cho, Yaeyama-gun, Okinawa, 907-1541 Japan; 4 United Graduate School of Agricultural Science, Kagoshima University, Kagoshima, Japan

**Keywords:** Biogeography, Phylogenetics

## Abstract

*Rhizophora* is a key genus for revealing the formation process of the pantropical distribution of mangroves. In this study, in order to fully understand the historical scenario of *Rhizophora* that achieved pantropical distribution, we conducted phylogeographic analyses based on nucleotide sequences of chloroplast and nuclear DNA as well as microsatellites for samples collected worldwide. Phylogenetic trees suggested the monophyly of each AEP and IWP lineages respectively except for *R. samoensis* and *R.* × *selala*. The divergence time between the two lineages was 10.6 million years ago on a dated phylogeny, and biogeographic stochastic mapping analyses supported these lineages separated following a vicariant event. These data suggested that the closure of the Tethys Seaway and the reduction in mangrove distribution followed by Mid-Miocene cooling were key factors that caused the linage diversification. Phylogeographic analyses also suggested the formation of the distinctive genetic structure at the AEP region across the American continents around Pliocene. Furthermore, long-distance trans-pacific dispersal occurred from the Pacific coast of American continents to the South Pacific and formed F1 hybrid, resulting in gene exchange between the IWP and AEP lineages after 11 million years of isolation. Considering the phylogeny and phylogeography with divergence time, a comprehensive picture of the historical scenario behind the pantropical distribution of *Rhizophora* is updated.

## Introduction

Mangrove forests are the most critical component of coastal and estuarine ecosystems covering over 150,000 km^2^ in 123 countries across the tropics and the subtropics^1^. They provide tremendous ecological services for human beings, however, deforestation of mangrove forests are ongoing worldwide by anthropogenic as well as climate changes^2,3^. Conservation of mangrove forests is becoming a global concern, and local, regional and nation-wide conservation activities are prominent in the world, however, our understandings on the global mangrove are limited because studying mangrove across nations and continents over pantropical distribution was difficult^4^.

The present pantropical distribution of mangrove plants could be due to the long-distance dispersal of their diaspores (seed, fruit, or propagule) by ocean currents^5^. Late Cretaceous fossil evidence of mangroves^6,7^ suggested the presence of mangrove over the world at that time. However, the evolutionary process and regional diversification of mangrove species to form the present pantropical distribution of mangrove forests are poorly understood. Uncovering the formation process of the pantropical distribution of mangroves will help deeper understandings of the global mangrove and provide us new insight into future changes of the mangrove ecosystem under global climate change.

Previous studies inferred a historical process of the pantropical distribution formation by considering the interactions between sea-dispersal and geological history based on the distribution patterns and fossil records of mangroves^5^. Duke et al.^8^, in particular, suggested constructing the phylogenetic history of a pantropical genus *Rhizophora* L. (Rhizophoraceae), which is a key taxon in revealing the global and disjunctive distribution patterns of mangroves. The genus *Rhizophora* dominates most of the mangrove forests in both Indo-West Pacific (IWP) and Atlantic-East Pacific (AEP) regions^9,10^, and the fossil records of the genus and family are relatively abundant owing to the distinct shape of the propagules. By studying the fossil records of the genus in detail, a scenario in which two lineages of *Rhizophora* diversified for IWP and AEP lineages owing to the closure of the Tethys Seaway in the Oligocene was proposed^5^. This scenario was the most comprehensive one at that time; however, it was merely a deduction based on fossil records and geological history.

Many mangrove researchers tried to understand the most intriguing question, that is, the historical process of formation of the world mangroves, focusing on the genus *Rhizophora* by using molecular markers^11–16^. Divergence times between the species in IWP and AEP regions have been estimated by using various molecular markers and sampling coverages. Lo et al.^14^ suggested that the divergence time between IWP and AEP lineages of *Rhizophora* was approximately 48 (± 3) Ma (million years ago) using cpDNA, ITS, and ISSR markers based on samples collected from a relatively wide range of distribution, however, they used limited numbers of AEP samples and no samples from West Africa. Chen et al.^15^ estimated the divergence time to be approximately 12.7 Ma (95% HPD (Highest Posterior Density), 10.4 Ma-15.5 Ma) using five nuclear genes based on less number of samples and used only two AEP samples from West Africa. Although it was not stated in the paper at all, a supplementary data of Xu et al.^16^ implied that the divergence time could be 10.8 Ma (95% credible interval 9.2 Ma – 12.8 Ma) using 590 single-copy genes extracted from genomic data based on only one representative sample from the AEP region. Even though Chen et al.^15^, as well as Xu et al.^16^ provided more reasonable divergence time of the two potential lineages of *Rhizophora* based on longer nucleotide sequences than any other previous studies, their study was based on only one or two representative samples from AEP *Rhizophora* species without showing the most critical basis for the global distribution, that is, the monophyly of AEP and IWP lineages respectively.

To understand the comprehensive picture of the phylogeographic process of *Rhizophora*, phylogenetic studies including all species as well as samples covering distinct lineages and wide distribution range are essential. Multiple samples from the wide distribution range of the AEP region are particularly important because the monophyly of the AEP lineages has not been clarified by nuclear DNA data, even though the presence of considerably distinct lineages across the American continents and West Africa was reported based on cpDNA and microsatellite data^13^. Numbers of distinct lineages were differentiated on both Pacific and Atlantic sides of the American continents, but none of the previous studies on the global phylogeny of *Rhizophora* included those distinct lineages in their phylogenetic analyses.

The sibling relationship between *Rhizophora samoensis* in South Pacific Islands in the IWP region and *Rhizophora mangle* in the AEP region has long been an enigmatic question discussed based on their morphological similarity^10^, and have recently been answered by molecular analyses using cpDNA sequences and microsatellite^13^. *Rhizophora samoensis* has an identical haplotype with the one of *R. mangle* in the Pacific coast of the American continents, which suggests at least a single long-distance migration occurred across the East Pacific. This unusual trans-pacific long-distance dispersal might have provided the opportunity for the two genetically distinct lineages, IWP and AEP lineages, to meet in the South Pacific Islands. Previous studies reported the presence of a putative hybrid species, *R.* × *selala*, between the two distinct lineages^12,14,17–19^, however, the phylogeographic process of the hybrid formation and hybridization patterns across the South Pacific Islands are still in question.

In this study, to obtain the most comprehensive scenario of historical processes that shaped the present pantropical distribution of mangrove forests, we performed the following analyses based on global sampling, including all species of *Rhizophora* as well as regional representative populations over the world. We performed 1) phylogenetic analyses using both cpDNA and nuclear DNA markers to obtain robust phylogeny to test the monophyly of the AEP lineage of *Rhizophora* in particular, 2) phylogeographic analyses to understand the historical process over the broad distribution range using the same molecular markers, and 3) estimation of divergence time by cpDNA phylogeny to give a robust time scale of mangrove diversification followed by ancestral range estimation to know the formation history of the distribution range. In addition, to understand the patterns of hybrid formation between AEP and IWP lineages of *Rhizophora* at the South Pacific. We also performed 4) population genetic analyses by comparing cpDNA and nuclear DNA sequences plus microsatellite genotypes. By incorporating the results obtained from all the above analyses, we propose an updated historical scenario of the formation process of the pantropical distribution of *Rhizophora.*

## Results

### Sequence variability

**Figure 1:**
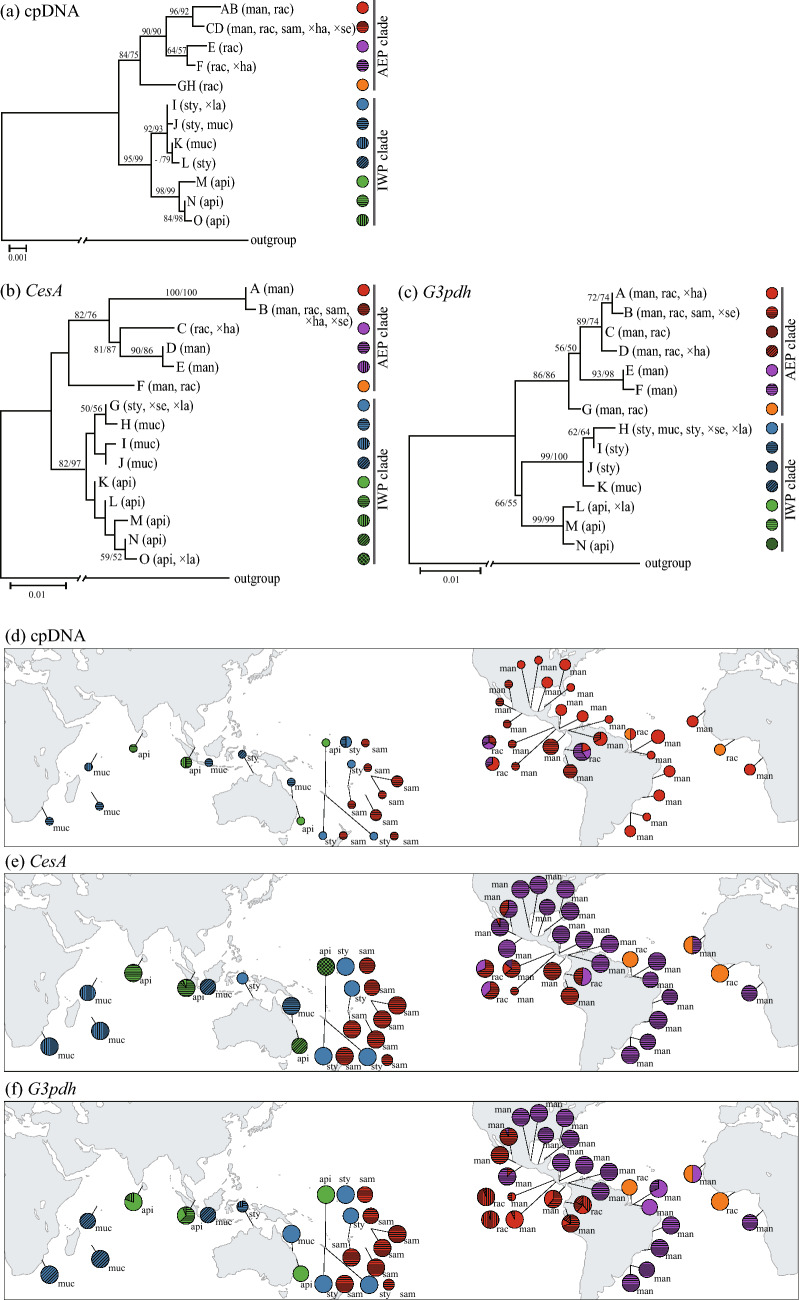
Trees of maximum parsimony topologies and geographical distributions of the haplotype/allele from combined chloroplast DNA sequences (**a**), and nuclear DNA sequences (**b**, *CesA* and **c**, *G3pdh*) of *Rhizophora* species. The ML analysis yield congruent MP topology (not shown). Numbers above branches indicate bootstrap probabilities greater than 50% for parsimony (left) and likelihood (right), respectively. Colored circles on the right of OTU indicate DNA sequences shown on the maps (**d–f**) indicating localities of sampled populations. Colored pie chart on the map indicate the frequency of chloroplast DNA haplotypes (**d**), and nuclear DNA alleles (**e**, *CesA* and** f**, *G3pdh*) of *Rhizophora* species except for hybrid species. Abbreviations in brackets in the tree and along pie chart on the map indicate species names: man, *R. mangle*; rac, *R. racemosa*; sam, *R. samoensis*; sty, *R. stylosa*; api, *R. apiculata*; muc, *R. mucronata*; × ha, *R.* × *harrisonii*, × la, *R.* × *lamarckii*, × se, *R.* × *selala*.

cpDNA–-The aligned sequences of the four cpDNA regions were 3912 bp containing 39 polymorphic sites, which produced 12 haplotypes in 89 samples of *Rhizophora* (Table 1, Table S1). Five and eight haplotypes were found in the AEP and IWP regions, respectively, and only the haplotype CD was shared between the two regions (Table S2, Fig. 1). The haplotype diversity was higher in IWP species than AEP species regardless of the inclusion or exclusion of *R. samoensis* and the three putative hybrid species from the analyses (Table 2). The nucleotide diversity and theta were higher in IWP species in all including analyses, but these values were similar in excluding analyses of *R. samoensis* and the putative hybrid species (Table 2).

**Table 1:** Localities of sampled populations of *Rhizophora* and *Bruguiera* species and sample size for chloroplast (*N*_CP_) and nuclear DNA (*N*_NUC_) analyses.

Taxon	Mangrove region	Oceanic region	Locality	Voucher	*N*_CP_	*N*_NUC_	Acronyms
*R. mangle*	AEP	Pacific	Panama: El Salado	TK01110601	4	9	MPE
			Ecuador: Jambeli	TK99072003	3	18	MEJ
			Mexico: San Blas	TK03111903	1	7	MMS
			Mexico: Oaxaca	TK03112201	1	8	MMO
			Mexico: Colima	TK08121403	1	6	MMM
			Costa Rica: Tivives, Puntarenas	TK07010903	1	1	MCT
			Costa Rica: Boca del Rio Damas, Puntarenas	TK07011001	1	7	MCB
		Atlantic	Panama: Miramar	TK01110706	3	9	MPM
			Panama: Galeta	TK01103101	1	14	MPG
			Brazil: Praia do Crispim, Pará	TK99120303	3	4	MBP
			Brazil: APA de Algodoal, Pará	TK05032501	1	8	MBA
			Brazil: Santa Catarina 2 km NE from Airport	TK05031901	1	4	MBS
			Brazil: Santa Catarina near the Centro	TK05031903	2	12	MBC
			Brazil: Rio de Janeiro	TK05032101	2	11	MBR
			Brazil: Pernambuco	KT05032701	2	4	MBE
			U.S.A: Florida	TK05121701	2	7	MUF
			Mexico: Laguna de Sontecomapan, Veracruz	TK03112503	2	4	MSS
			Mexico: Santa Ana, Veracruz	TK03112601	2	7	MAL
			Mexico: Tonalá river, Veracruz-Tabasco	TK08120906	1	6	MML
			Mexico: Campeche	TK03112405	2	8	MMV
			Costa Rica: Moin, Limon	TK07011201	1	7	MMT
			Costa Rica: Laguna Gandoca, Limon	TK07011301	1	7	MMC
			Senegal: South of Mbour	TK00120301	2	8	MCM
			Angola: Luanda	TK01050601	2	4	MCL
*R. racemosa*	AEP	Pacific	Costa Rica: Tivives, Puntarenas	TK07010902	3	16	RCT
			Costa Rica: Boca del Rio Damas, Puntarenas	TK07011002	3	12	RCB
			Ecuador: Esmeraldas	TK02012204	5	18	REE
		Atlantic	Brazil: Mosquiero, Pará	TK05032602	2	4	RBM
			Ghana: Ankobra	KT04092402	2	11	RGA
*R. samoensis*	IWP	Pacific	Samoa: Satatoa	TK02102803	1	9	SSS
			Samoa: Safata	TK02102608	2	7	SST
			Tonga: Sopu	TK02102401	2	7	STS
			New Caledonia: Canala	TK07092701	1	4	SNC
			New Caledonia: Yate	TK07092804	1	7	SNY
			New Caledonia: Nouméa	TK07092901	1	2	SNN
			Fiji: Muanikau	KT09012606	1	4	SFM
			Fiji: Vunda Junction	KT09012904	1	8	SFV
*R. stylosa*	IWP	Pacific	New Caledonia: Canala	TK07092705	2	5	TNC
			New Caledonia: Yate	TK07092805	1	8	TNY
			New Caledonia: Nouméa	TK07092902	1	7	TNN
			Fiji: Muanikau	KT09012607	1	4	TFM
			Australia: Darwin	TK04121001	1	2	TAD
*R. mucronata*	IWP	Pacific	Australia: Sarina beach	TK04121502	1	6	UAS
		Indian Ocean	Singapore: Sungei Buloh	TK04121804	1	4	USS
			Seychelles: Port Glaud	KT05090601	1	4	USP
			Mauritius: Mahébourg	KT05090301	1	5	UMM
			South Africa: Beach Wood	KT04091901	1	5	UBW
*R. apiculata*	IWP	Pacific	New Caledonia: Canala	TK07092703	1	6	ANC
			Australia: Centenial Lakes	TK04121203	1	4	AAC
		Indian Ocean	Singapore: Sungei Buloh	TK04121803	2	8	ASS
			Sri Lanka: Waikkal	TK04110701	1	5	ASW
*R.* × *harrisonii*	AEP	Pacific	Costa Rica: Boca del Rio Damas, Puntarenas	TK07011003	2	6	HCB
*R.* × *lamarckii*	IWP	Pacific	New Caledonia: Canala	TK07092702	1	9	LNC
*R.* × *selala*	IWP	Pacific	New Caledonia: Canala	TK07092704	1	11	ENC
			New Caledonia: Yate	TK07092801	1	1	ENY
			Fiji: Lautoka	KT09012902	1	8	EFL
*Bruguiera gymnorrhiza*	IWP	Pacific	Australia: Centenial Lakes	TK04121201	1	1	BAC
Total					90	398

**Table 2:** Genetic diversity parameters estimated by chloroplast and nuclear DNA sequences.

Taxon	cpDNA						*CesA*					*G3pdh*				
	*N*_P_	*N*	*H*	*Hd*	*Pi*	*θ*	*N*	*H*	*Hd*	*Pi*	*θ*	*N*	*H*	*Hd*	*Pi*	*θ*
*R. mangle*	24	42	2	0.438	0.0009	0.0005	180	5	0.375	0.0122	0.0078	180	7	0.556	0.0054	0.0030
*R. racemosa*	5	15	5	0.829	0.0027	0.0021	61	3	0.652	0.0222	0.0094	61	5	0.629	0.0032	0.0021
*R. samoensis*	8	10	1	0.000	0.0000	0.0000	48	1	0.000	0.0000	0.0000	48	1	0.000	0.0000	0.0000
*R. stylosa*	5	6	3	0.600	0.0003	0.0004	26	1	0.000	0.0000	0.0000	26	3	0.147	0.0006	0.0010
*R. mucronata*	5	5	2	0.400	0.0001	0.0001	24	3	0.582	0.0023	0.0012	24	2	0.383	0.0024	0.0014
*R. apiculata*	4	5	3	0.800	0.0008	0.0007	23	5	0.766	0.0051	0.0024	23	3	0.426	0.0020	0.0014
*R.* × *harrisonii*	1	2	2	1.000	0.0033	0.0033	6	2	0.485	0.0156	0.0106	6	2	0.530	0.0085	0.0053
*R.* × *lamarckii*	1	1	1	0.000	0.0000	0.0000	9	2	0.529	0.0047	0.0026	9	2	0.529	0.0099	0.0054
*R.* × *selala*	1	3	1	0.000	0.0000	0.0000	20	2	0.513	0.0192	0.0088	20	2	0.513	0.0156	0.0071
AEP	30	59	5	0.655	0.0014	0.0015	247	6	0.604	0.0181	0.00851	247	7	0.728	0.0064	0.00283
IWP	24	30	8	0.733	0.0039	0.0026	150	8	0.751	0.0199	0.00741	150	8	0.729	0.0162	0.00637
All	54	89	12	0.763	0.0029	0.0024	397	13	0.763	0.0237	0.01045	397	14	0.830	0.0134	0.00684
AEP*	29	57	5	0.643	0.0014	0.0015	241	6	0.595	0.0178	0.0085	241	7	0.720	0.0064	0.0028
IWP*	14	16	7	0.750	0.0015	0.0013	73	7	0.769	0.0045	0.0023	73	7	0.717	0.0094	0.0045
All*	43	73	12	0.763	0.0029	0.0025	314	13	0.749	0.0222	0.0108	314	14	0.820	0.0129	0.0071
* excluding *R. samoensis* and putative hybrid species																

*N*_P_, number of populations; *N*, number of individuals; *H*, number of haplotypes or alleles, *Hd*, haplotype (allele) diversity; *Pi*, nucleotide diversity; θ, number of mutation per site.

*CesA*–-The aligned sequences of the *CesA* were 555 bp long containing 42 polymorphic sites, which produced 15 alleles in 397 samples of *Rhizophora* (Table 1, Table S1). Six and ten alleles were found in AEP and IWP species, respectively (Table S2, Fig. 1). The allelic diversity was higher in IWP species, but the nucleotide diversity and theta tended to be higher in AEP species in both analyses (Table 2).

*G3pdh*–-The aligned sequences of the *G3pdh* were 668 bp, there were, however, a ca. 60 bp ambiguously aligned region because of poly T and C complex. Therefore, we excluded the complex region for further analysis. As a result, 608 bp was aligned in *G3pdh* containing 31 polymorphic sites, which produced 14 alleles in 397 samples of *Rhizophora* (Table 1, Table S1). Seven and eight alleles were found in AEP and IWP species, respectively (Table S2, Fig. 1). The allelic diversity was similar in AEP and IWP species, and the nucleotide diversity was higher in IWP species in both the analyses (Table 2).

### Phylogenetic relationships

FindModel identified the best model for each gene: GTR + gamma for cpDNA, Tamura-Nei + gamma for *CesA*, and Hasegawa-Kishino-Yano + gamma for *G3pdh*. The results of MP and ML showed congruent topology for the monophyly of each AEP and IWP species except for *R. samoensis* and *R.* × *selala* (Fig. 1). *Rhizophora samoensis* had an identical haplotype and allele with AEP species clade, and *R.* × *selala* had the same cpDNA haplotype with AEP species and same nuclear alleles with the AEP and IWP clades in hetero. *Rhizophora mangle* and *R. racemosa* G. Mey. did not make its own clade within the AEP clade. *Rhizophora apiculata* Blume formed a monophyletic clade with *R.* × *lamarckii* Montrouz., and *R. stylosa* Griff. and *R. mucronata* Lam. formed a monophyletic clade together in the IWP clade.

### Minimum spanning network of haplotypes

All three Minimum Spanning Networks constructed using haplotypes of cpDNA, *CesA,* and *G3pdh* showed clear separation of IWA and AEP groups (Fig. S2). A haplotype possessed by *Rhizophora* species (colored in orange: GH in cpDNA, F in *CesA*, G in *G3pdh*) was found only from the Atlantic region and was placed at the basal position of the AEP group in each network.

### Global population genetic structure

Global distribution of cpDNA haplotype and nuclear allele in the six non-putative hybrid species are shown in Fig. 1. The details of haplotype and allele frequency are in Table S2. The patterns of nuclear alleles in putative hybrid species were shown in Table 3 and Table S3. There are only one cpDNA haplotype (CD) and *CesA* and *G3pdh* alleles (both B) which can be observed in both AEP and IWP species. These haplotypes and alleles were commonly and geographically-widely found in the species in the AEP region; however, they were found only in *R. samoensis* and *R.* × *selala* in the IWP region.

**Table 3:** Localities of sampled populations of *Rhizophora samoensis*, *R. stylosa,* and *R.* × *selala* in New Caledonia and Fiji, and sample size for PCR–RFLP and microsatellite analyses.

Locality	Taxon	Voucher	*N*
New Caledonia: Canala	*R. stylosa*	TK07092705	8
	*R.* × *selala*	TK07092704	8
	*R. samoensis*	TK07092701	7
New Caledonia: Yate	*R. stylosa*	TK07092805	15
	*R.* × *selala*	TK07092801	8
	*R. samoensis*	TK07092804	10
New Caledonia: Nouméa	*R. stylosa*	TK07092902	16
	*R. samoensis*	TK07092901	5
Fiji: Muanikau*	*R. stylosa*	TK09012607	6
	*R. samoensis*	KT09012606	17
Fiji: Bau	*R. stylosa*	TK09012702	8
	*R. samoensis*	KT09012701	22
Fiji: Lautoka	*R. stylosa*	TK09012903	19
	*R.* × *selala*	KT09012902	1
	*R. samoensis*	KT09012901	15
Total			165

*34 and 30 hypocotyls were collected in *R. samoensis* and *R. stylosa*, respectively.

In the AEP region, each haplotype and allele were widely distributed. A remarkable genetic differentiation between the Pacific and the Atlantic side were found in cpDNA haplotypes, although there are few overlaps between the two regions in terms of both nuclear DNA alleles. Populations on the Pacific coast in Mexico and Costa Rica had the alleles which widely dominant in the Atlantic region. In the IWP region, there are several haplotypes and alleles which were widely distributed or unique to a single locality.

### Divergence times

**Figure 2:**
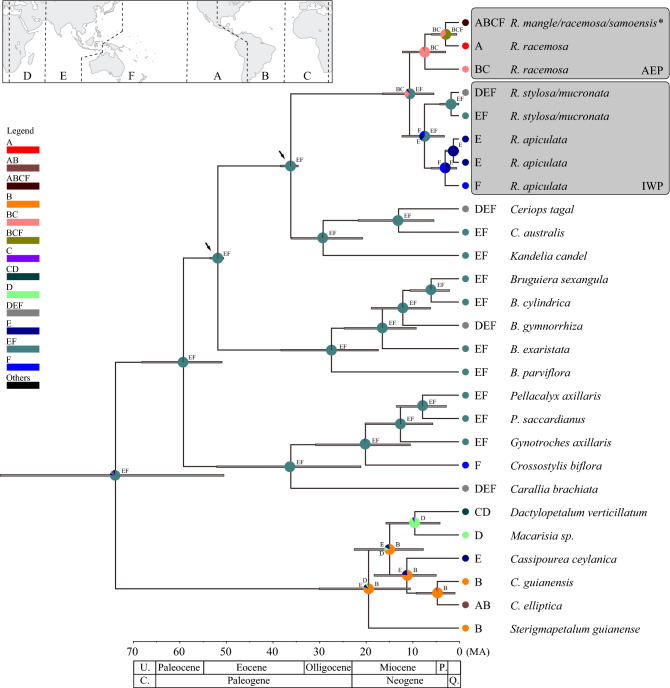
Chronograph and biogeographic history for *Rhizophora* and outgroup taxa based on the relaxed-clock Bayesian MCMC methods in BEAST and BioGeoBEARS in RASP using chloroplast DNA sequences. Error bars on nodes indicate 95% highest posterior densities around the mean dates. Arrows indicate fossil calibration points described in Methods. Probability of ancestral states are shown in pie chart. *Rhizophora samoensis is distributed in the IWP region but has an identical haplotype with AEP species.

To estimate divergence time for deeper nodes within *Rhizophora*, we conducted phylogenetic analyses with two selected cpDNA regions (*rbcL* and *atpB*-*rbcL* intergenic region) and two nuclear genes combining with the available data set deposited in DDBJ (DNA Data Bank of Japan). The most recent common ancestor of *Rhizophora* and sister mangrove genera, *Ceriops* Arn. and *Kandelia* (DC.) Wight et Arnott, was estimated to be 36.0 Ma (95% HPD 34.5–38.2 Ma: Fig. 2) by using cpDNA data including 27 OTUs gathered in the Bayesian analysis with the two calibration points. The split between AEP and IWP clades of *Rhizophora* was estimated to be 10.6 Ma (5.4–16.3 Ma). In the AEP clade, the split between *R. racemosa* and *R. mangle/R. racemosa/R. samoensis* clades were estimated to be 3.0 Ma (0.5–6.0 Ma) in the cpDNA analysis. In the IWP clade, the split between *R. apiculata* and *R. stylosa/R. mucronata* clades were estimated to be 7.4 Ma (3.1–12.2 Ma). The estimation time of the split between AEP and IWP clades estimated using the first calibration point only was 10.3 – 34.0 Ma (2.2 – 53.7 Ma) depending on the data set (Table S4).

### Biogeographic inference

Biogeographic stochastic mapping analyses in BioGeoBEARS yielded the BAYAREA + J model as the best- fitting model for the phylogeny of all Rhizophoraceae species (Table S5). The ancestral ranges of IWP and AEP were Indo-Malesia and Australasia (EF) (ML probability = 0.60) and East America and West Africa (BC) (ML probability = 0.26) (Fig. 2). The estimated ancestral range of all AEP species was East America and West Africa (BC) (ML probability = 0.92) (Fig. 2).

### Hybridization patterns in the South Pacific Islands

**Figure 3:**
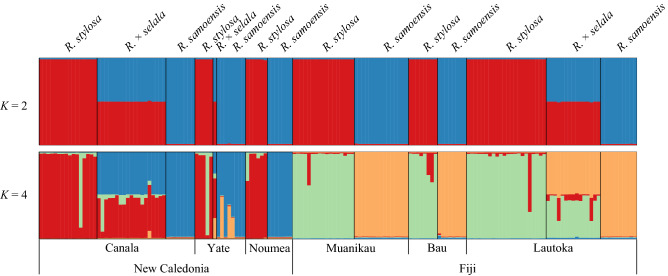
Results of STRUCTURE of *Rhizophora* species in the South Pacific Islands. Vertical columns represent individual plants, and the heights of bars of each color are proportional to the posterior means of estimated admixture proportions. The number of cluster *K* = 2 and *K* = 4 are shown.

The results of PCR–RFLP showed that all individuals of *R. stylosa* and *R. samoensis* including their hypocotyls possessed their specific genotypes in cpDNA and nuclear DNA, and all individuals of *R.* × *selala* possessed a particular combination of cpDNA haplotype, *R. samoensis* genotype, and nuclear DNA genotypes, *R. stylosa* and *R. samoensis* in hetero, which can be expected of F1 hybrid genotype (Tables S2 & S3). All propagules had the same genotypes as their mother tree of *R. stylosa* or *R. samoensis*. The results of STRUCTURE based on microsatellite data were shown in Fig. 3 and Fig. S1. The value of log-probability began to plateau at *K* = 4 and the highest delta *K* value was returned at *K* = 2 so that we showed *K* = 2 and *K* = 4 as most probable numbers of the cluster. In *K* = 2, the two clusters represented *R. stylosa* and *R. samoensis*, respectively, and both clusters can be admixed fifty-fifty in three populations of *R.* × *selala*. In *K* = 4, each of the two clusters represented *R. stylosa* or *R. samoensis* mostly depending on the locality. Red and green clusters were found in *R. stylosa* from New Caledonia or Fiji, and blue and orange ones in *R. samoensis*. Two clusters found in each locality were admixed in *R.* × *selala*.

## Discussion

### Phylogeny and timing of diversification of *Rhizophora*

This study provided the most comprehensive phylogeny that explains the present pantropical distribution of a mangrove genus *Rhizophora*. We included distinct lineages from a wide range of the AEP region and showed that all AEP samples were in the AEP clade that is sister to the IWP clade in all analyses using multiple DNA markers (Fig. 1). Although trans-oceanic dispersal and frequent hybridization have been reported in the genus *Rhizophora*, this result ensures an adequate discussion of the divergence time between the AEP and IWP lineages of *Rhizophora* based on the phylogenetic tree.

The divergence time between the AEP and IWP lineages was 10.6 Ma by using cpDNA and broad outgroup taxa (Fig. 2) and 11.0–11.5 Ma by a combination of three different regions (cpDNA, *CesA*, and *G3pdh*) (Table S4) which are similar to the ones, 12.7 Ma by five nuclear genes^15^ and 10.8 Ma by 590 coding genes^16^ and far different from 48 (± 3) Ma by cpDNA and ITS^14^. Because our study based on global sampling showed the monophyly of the AEP lineage and similar estimates for the divergence time between the IWP and AEP linages, we consider that the estimates of Xu et al.^16^ also can be reasonable even though they used only one sample from the AEP region.

The separation of the AEP and IWP lineages reasonably coincide with the closure of the Tethys Seaway. The biogeographic stochastic mapping analyses suggested that the common ancestor of the AEP and IWP lineages was separated into two ancestral ranges 10.6 Ma: the Atlantic region (BC) for the AEP lineage, and the Indo-Pacific region (E, F, and EF) for the IWP linage (Fig. 2). The separation can be attributed to the formation of the land barrier at Tethys Seaway that separated the Atlantic and Indo-Pacific regions around 11 to 20 Ma^20–24^. The common ancestor of the AEP lineage at the Atlantic (BC) was suggested to expand the range to the Pacific regions (A and ABCF) in the later diversification (Fig. 2). Although there were only three extant AEP lineages of *Rhizophora* in the dated tree (Fig. 2) due to less length of sequence data, longer length of cpDNA sequence showed the presence of five haplotypes (AB, CD, E, F, and GH) (Fig. 1a, Fig. S2), and three haplotypes (CD, E, and F) were exclusively found in the Pacific region (Fig. 1a,d). This remarkable segregation between the Pacific and Atlantic populations can be attributed to the closure of the Isthmus of Panama at 3 Ma^25^ after the range expansion of *Rhizophora* species from Atlantic to the Pacific.

Our results provided clear evidence of the recent trans-pacific migration of *R. mangle* from the Pacific side of the New World (AEP) to the South Pacific Islands (IWP)^13,14^. The haplotype possessed by *R. samoensis* from the South Pacific Islands and *R. mangle* from the Pacific side of the New World was identical both in cpDNA (CD) and nuclear DNA markers (B in both markers) (Fig. 1). Our data also confirmed that *R.* × *selala* in the South Pacific Islands was a F1 hybrid asymmetrically formed by *R. stylosa* as father lineage and *R. samoensis* as mother lineage. In addition, our microsatellite data suggested that independent events of hybrid formation occurred recurrently in New Caledonia and Fiji (Fig. 3). These results suggest that *R.* × *selala* are F1 hybrids between the species from IWP and AEP lineages. Although hybrids are recurrently formed when the two lineages meet, they are sterile F1 hybrids because of strong reproductive isolation caused by long separation of parental lineages for about 11 Ma. Our results supported the previous observation that *R.* × *selala* had low pollen fertility and abnormal tetrads^18,19^.

### Updated scenario for the formation of the distribution pattern of *Rhizophora*

**Figure 4:**
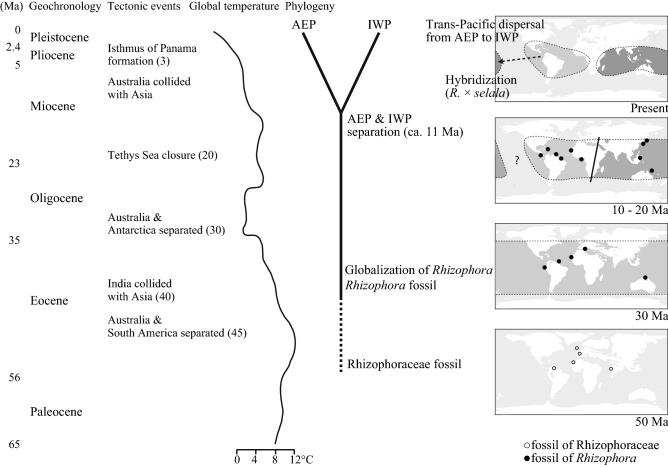
Historical scenario of diversification of *Rhizophora*. Concurrent significant tectonic events, global temperature, and fossil occurrences have been listed for comparison with the result of phylogenetic analysis in this study. The patterns of global temperature and fossil occurrences were modified from previous studies^7,30^.

According to our findings and some previous studies, we finally updated the historical scenario behind the formation of the distribution pattern of the mangrove genus *Rhizophora,* proposed by Duke et al.^5^ We illustrated a synthetic scenario of the historical process with the tectonic events and changes of global temperature that shape the present pantropical distribution of the mangrove genus *Rhizophora* in Fig. 4 and as followings:

#### Eocene—Oligocene (50–23 Ma)

*Rhizophora* originated during the Eocene and was distributed worldwide by the Middle and Late Eocene evidenced by several comprehensive assessments of fossil records^6,7,26–29^. Our biogeographic analysis supported that the origins of extant *Rhizophora* were mostly in IWP region and further spread to AEP region (Fig. 2). The mangrove habitat might have been more widely extended in longitude than at present, because the average temperature of the earth was 4–12 °C higher^30^. In fact, the pollen of mangrove palm, *Nypa*, was reported from the Early Eocene layer in Tasmania^31^ and New Zealand^32^, where no mangrove habitat occurs at present. The wider longitudinal distribution area of mangrove might be achieved by the migration of propagules in the east–west direction between the Old World and New World, via the southern route around the African continent. Furthermore, ocean currents through “Tethys Seaways (Mediterranean Sea)” also could work as a dispersal corridor between the two regions (Fig. 4, "Globalization of *Rhizophora*").

#### Miocene (23–10 Ma)

By geological approaches, the closure of Tethys Seaways was estimated from 11 to 20 Ma^20–24^ which is consistent with our estimation of the divergence time ca. 11 Ma for the deep phylogenetic break between IWP and AEP lineages. The closure of the Tethys Seaway created a land barrier between the IWP and AEP regions^33^. In addition, Mid-Miocene cooling occurred at the same time^30^ would narrower the distribution range to the equator and prevented the migration of propagules via the southern route around the African continent (Fig. 4, "IWP & AEP separation").

#### Late Miocene to Present (10 Ma-)

The global cooling at Late Miocene and Pleistocene might have promoted a decrease in the population size for many mangrove species via sea level drop, and separation to regional populations may lead allopatric speciation in *Rhizophora*. In the AEP region, both *R. mangle* and *R. racemosa* expanded their distribution across the American continents from the Atlantic region via migration through the Panama Seaway. After the closure of the Panama Seaway at 3 Ma^25^, genetic differentiation between the Pacific and Atlantic populations of both species occurred. In the IWP region, the *R. apiculate, R. stylosa*, and *R. mucronata* might form their present distribution through different demographic histories in each species despite having sympatric distributions today^34,35^. Finally, eventual trans-Pacific dispersal of *R. mangle* happened from American continents to the South Pacific. The extremely long-distance dispersal caused secondary contact between distinct lineages of *Rhizophora*, which can independently produce the sterile F1 offspring in the South Pacific Islands (Fig. 4, "Trans-Pacific dispersal from AEP to IWP").

## Methods

### Plant collections

We collected leaf samples from 56 populations of all eight species including three putative hybrid species across the IWP and AEP regions for phylogenetic analyses (Table 1). Upon collection, leaf samples for DNA extraction were put in reclosable plastic bags and dried with silica gel. We also collected 15 populations of *R. stylosa*, *R. samoensis*, and *R.* × *selala* in New Caledonia and Fiji for investigation of hybridization pattern in the South Pacific Islands (Table 3). In addition, a part of the hypocotyl of propagule for DNA extraction was also collected from *R. samoensis* and *R. stylosa* in Fiji (Table 3). Twenty-nine populations from the AEP region and nine populations form the IWP region using in this study are the same as those used in a previous study^13^. Identification of the species was done following the keys in Tomlinson^10^ and at least one voucher specimen from each population and species was deposited in the Herbarium at the Herbarium of University of Ryukyus (URO).

### DNA extraction and sequencing

Genomic DNA was extracted from the dried leaf and hypocotyl tissue using the cetyltrimthyl ammonium bromide (CTAB) extraction method^36^ and purified using GENECLEAN III Kit (MP Biomedicals, Solon, USA). PCR amplification and sequencing were performed using 1 to 18 individuals from each population (Table 1). Sequences of four cpDNA regions, *atpB-rbcL* intergenic spacer (IGS), *trnS-trnG*, *rpl16* intron, and *rbcL* gene, were determined with the same protocol as in the previous study^13^. Other available sequences from Rhizophoraceae were obtained from GenBank accessions (Table S1). In addition, a part of *rpl16* intron sequence was determined in 165 individuals of *R. stylosa*, *R. samoensis*, and *R.* × *selala*, and 64 hypocotyls of the propagule of *R. stylosa* and *R. samoensis* in New Caledonia and Fiji. Two nuclear DNA regions, glyceraldehyde 3 phosphate dehydrogenase (*G3PDH*), and cellulose synthase (*CesA*) homolog were amplified using primers reported in Strand et al.^37^ and Cronn et al.^38^. Sequencing reaction was performed with using primers GPDX7F^37^ and G3PDH-788R1 (5′-CAATGAAGTCTGTGGATACCAA-3′) for *G3PDH*, and CesA-1150F (5′-CCACCTGAGCAGCAGATGGAAG-3′) and CesA-1800R (5′-ACGACAGTTGAAAGTGGCTGTGC-3′) for *CesA*. In cases where the phenogram of the two nuclear DNA regions showed duplicated peaks in more than two sites, we segregate the alleles of the PCR amplicon with single-strand conformation polymorphism (SSCP) analyses^39^ and re-sequenced after acrylamide gel extraction.

### Phylogenetic inference

Genetic diversity parameters were estimated using DnaSP 5.10^40^. Phylogenetic analyses were performed using the Wagner maximum parsimony (MP) and Maximum Likelihood (ML) methods based on three different matrices, the combined sequence data of four cpDNA, *G3PDH* and *C3esA*, separately. *Bruguiera gymnorrhiza* was used as outgroup for all of the *Rhizophora* species according to previous phylogenetic study^11^. MP and ML analyses were conducted using PAUP*^41^ and RAxML^42^, respectively. For the ML analyses, FindModel (http://hcv.lanl.gov/content/hcvdb/findmodel/) was used to find the best fitting model for nucleotide sequence evolution.

### Minimum spanning network

We used PopART^43^ to construct a minimum spanning network^44^ using the alignments of haplotype and allele sequences of cpDNA, *G3PDH* and *C3esA*.

### Divergence time estimates

To test temporal aspects of phylogeographic breaks, we inferred divergence times using a relaxed-clock method in the program BEAST v1.6.2^45^. Two chloroplast regions, *rbcL* and *atpB*-*rbcL*, were used to conduct comprehensive analyses by including compatible data from other genera in Rhizophoraceae in previous phylogenetic study^11^. In addition, two nuclear regions, *CesA* and *G3pdh* obtained in this study were used for the dating analyses. We estimated divergent times using 12 different combinations of data and taxon because the nucleotide diversity and available sample of the outgroup taxa were different depending on the DNA regions. As previously reported^13,14^, *Rhizophora* species had experienced hybridization and shared haplotype/genotype partially, hence the sorting of haplotypes/genotypes into a sample increased the number of OTUs which shared identical haplotype or genotype. We first conducted a multispecies coalescent analysis^46^ in the dating analyses using the combination of different genes. The multispecies coalescent analysis estimates a species (taxon) tree based on unlinked multi-locus sequence data, taking into account that gene trees are incorporated in a shared species (taxon) tree by following the stochastic coalescent process. For the analysis that included multiple regions, three OTUs from *Rhizophora* samples, such as *R. stylosa*—*R. mucronata*, *R. apiculata*, and *R. mangle* – *R. racemosa* were used for the coalescent analysis. The analysis was conducted using the program BEAST v1.6.2^45^. We used the fossil record to place priors on the crown age of a node, arising of mangrove clade (*Bruguiera* Lam., *Kandelia*, *Ceriops*, and *Rhizophora*) in Rhizophoraceae. In the review of mangrove fossil record^7^, fruits and pollen of ancestors very close to modern mangrove genera in Rhizophoraceae existed in the Early Eocene (50 Ma), but not in the Palaeocene (55 Ma) when the fossil record of another mangrove genus, *Nypa,* is abundant worldwide. Therefore, we set a lognormal prior offset of 50 Ma with a mean and standard deviation of 0.5 for the first calibration point. In addition, ancestral *Rhizophora* fossil has been reported to the late Eocene (33.9–38 Mya)^47^. We set a lognormal prior offset of 34 Ma with a mean and standard deviation of 0.5 for crown age of a node of a monophyly clade of *Ceriops*, *Kandelia*, and *Rhizophora* for the second calibration point. We used the two calibration points for the cpDNA analyses using broad species in Rhizophoraceae. On the other hand, we only used the first calibration point for 12 different combinations of data and taxon due to the limitation of the taxon coverage to use the second calibration point. We conducted three independent searches of 10 × 10^7^ generations sampling every 1000 generations under the models selected by FindModel in each genetic region. The results log files from the three runs were combined by the program LogCombiner v1.7.5^45^. We confirmed that ESS values of all estimated parameters after 20% burn-in were > 200 in the program Tracer v1.5^45^. We also generate a maximum clade credibility tree using 20% burn-in trees obtained in three runs by LogCombiner v1.7.5 and TreeAnnotator v1.7.5^45^ and presented the mean and 95% highest posterior density (HPD) of node ages by FigTree v1.4^48^.

### Biogeographic inference

To infer the ancestral range for *Rhizophora*, we used the ML method implement in BioGeoBEARS 0.2.1^49^ in the RASP 4.2 package^50^. We defined the geographical ranges as A = West America, B = East America, C = West Africa, D = East Africa, E = Ind-Malesia, and F = Australasia according to the previously reported definitions of geographic ranges of *Rhizophora*^5^. We estimated ancestral ranges and biogeographical events on the BEAST MCC trees of Rhizophoraceae species based on two cpDNA regions (Fig. 2), using a likelihood-based framework in BioGeoBEARS that allows for testing various biogeographical models. The best model was selected using AIC corrected for sample size (AICc). We rerun the BioGeoBEARS analysis on 100 randomly sampled post-burn- in BEAST chronograms. The number of maximum areas per ancestral range was set to four.

### PCR–RFLP and microsatellite analysis

To reveal hybridization patterns in the South Pacific Island, PCR–RFLP and microsatellite genotyping were conducted in 165 individuals of *R.* × *selala*, *R. samoensis*, and *R. stylosa* in New Caledonia and Fiji, and 64 hypocotyls of propagule of *R. samoensis* and *R. stylosa* in Fiji (PCR–RFLP only). For PCR–RFLP, the amplified *G3PDH* and *CesA* fragments in the 165 individuals and 64 hypocotyls were digested with restriction enzymes, *MspI* (CCGG) and *Sau3A* (GATC), respectively. Based on the sequence data, we expected the length of DNA fragments of *R. stylosa* after this treatment to be ca. 10, 30, 670 bp in *G3PDH* and 50, 50, 70, 390 bp in *CesA*, and those of *R. samoensis* to be 10, 700 bp and 50, 70, 440 bp. Digested DNA fragments were detected 2% agarose electrophoresis, and determined genotypes of two nuclear DNA regions. In addition, four microsatellite markers (RM50, RS19, RS33, and RS59) developed by Takayama et al*.*^51,52^ were selected for the analysis of hybridization patterns in New Caledonia and Fiji, because the four markers can be constantly amplified in both AEP and IWP species. We determined microsatellite genotypes in 165 individuals of *R.* × *selala*, *R. samoensis*, and *R. stylosa* in New Caledonia and Fiji. PCR amplification and genotyping were performed according to the previous study^13^. Genetic structure was evaluated by the Bayesian clustering method using STRUCTURE 2.3.3^53–55^. Markov chain Monte Carlo (MCMC) searches consisted of 100,000 “burn-in” steps, followed by 100,000 iterations. Twenty replicate runs were performed at each *K* from 1 to 10 and calculated the log-probability and the delta *K* proposed by Evanno et al.^56^, given a certain value of *K* for seeking the best-fit *K* value for the data.

## Supplementary Information

Supplementary Information

## Data Availability

All data sets are provided in the Supplementary Information and deposited in DDBJ.
