# Supplementary material for: Global phylogeography of a pantropical mangrove genus Rhizophora
Source: Sci Rep. 2021 Mar 30;11:7228. doi: 10.1038/s41598-021-85844-9 (PMC8009884; doi:10.1038/s41598-021-85844-9)
Supplement: Supplementary file 1 — Supplementary Information [file 41598_2021_85844_MOESM1_ESM.docx]

***Supplementary Information***

***Title:*** Global phylogeography of a pantropical mangrove genus *Rhizophora*

***Author:*** Koji Takayama^1*^, Yoichi Tateishi^2^, Tadashi Kajita^3*^

***Author affiliation:*** ^1^ Department of Botany, Graduate School of Science, Kyoto University, Kitashirakawa Oiwake-cho, Sakyo-ku, Kyoto 606-8502, Japan

^2^ Faculty of Education, University of The Ryukyus, Senbaru 1, Nakagami-gun, Okinawa 903-0129, Japan

^3^ Iriomote Station, Tropical Biosphere Research Center, University of the Ryukyus, 870 Uehara, Taketomi-cho, Yaeyama-gun, Okinawa, 907-1541, Japan.

***Corresponding authors:*** Koji Takayama (e-mail: takayama@sys.bot.kyoto-u.ac.jp), Tadashi Kajita (e-mail: kajita@mail.ryudai.jp)

Fig. S1. Results of STRUCTURE of *Rhizophora* species in South Pacific Islands. The open plots give the mean loge Pr(*X*|*K*) and standard deviation over 20 runs for each value of *K*. The solid plots give delta *K* showing a peak at the uppermost level of structure at the true value of *K*.


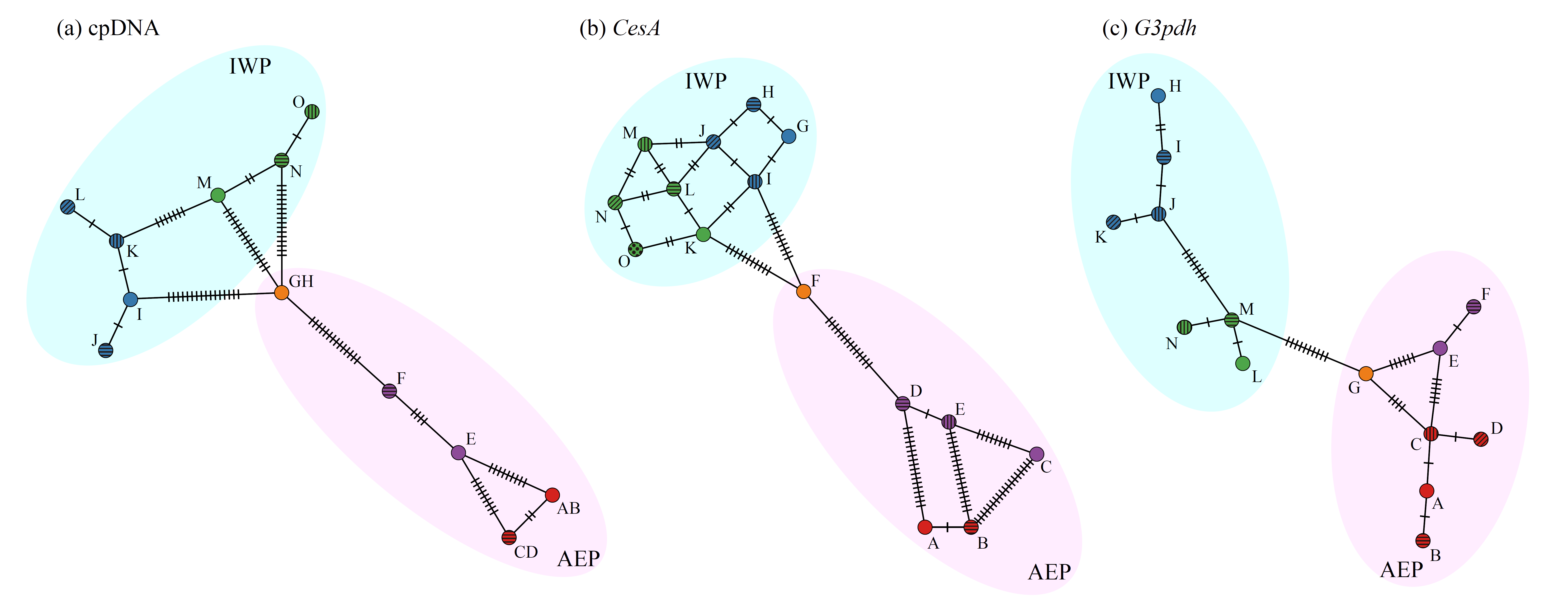


Fig. S2. Minimum spanning networks of the haplotype/allele of *Rhizophora* species based on the combined chloroplast DNA sequences (a), and nuclear DNA sequences (b, *CesA* and c, *G3pdh*). Bars on branches indicate nucleotide substitutions. Color patterns of haplotype/allele and letters correspond to those presented in Fig. 1.
